# Supplementary material for: Synergistic Effect between Zr-MOF and Phosphomolybdic Acid with the Promotion of TiF4 Template
Source: Molecules. 2020 Oct 13;25(20):4673. doi: 10.3390/molecules25204673 (PMC7587353; doi:10.3390/molecules25204673)
Supplement: Supplementary file 1 [file molecules-25-04673-s001.pdf]

Supporting Information

TiF_4 template promotes strong metal-support interactions (SMSI) between Zr-MOF and phosphomolybdic acid

Zhu Ding, Xiao Min Zhang, Xue Chang, Shuo Wang, Dan Hong Wang*,Ming Hui Zhang, Tian Hao Zhang*

Zhu Ding, Xue Chang, Shuo Wang, Prof. Dan Hong Wang*,

TKL of Metal and Molecule Based Material Chemistry, National Institute for Advanced Materials, School of Materials Science and Engineering, Nankai University, Tianjin 300350, China.

E-mail: dhwang@nankai.edu.cn

Xiao Min Zhang, Prof. Ming Hui Zhang

Key Laboratory of Advanced Energy Materials Chemistry (Ministry of Education), College of Chemistry, Nankai University, Tianjin 300071, China.

Prof. Tian Hao Zhang

School of physics, Tianjin Key Laboratory of Photonics Materials and Technology for Information Science, Nankai University, Tianjin 300350, China.

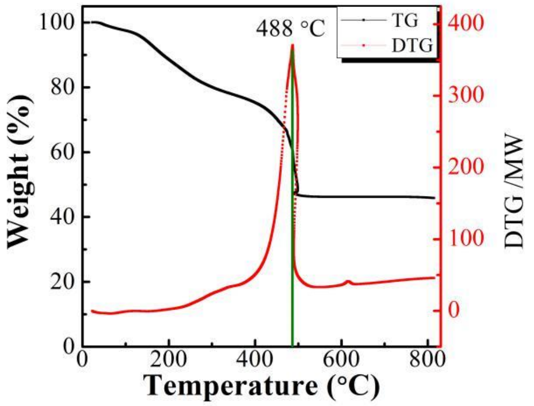

Fig. S1 TG curves in an N_2 atmosphere for TiF_4-PU precursor.

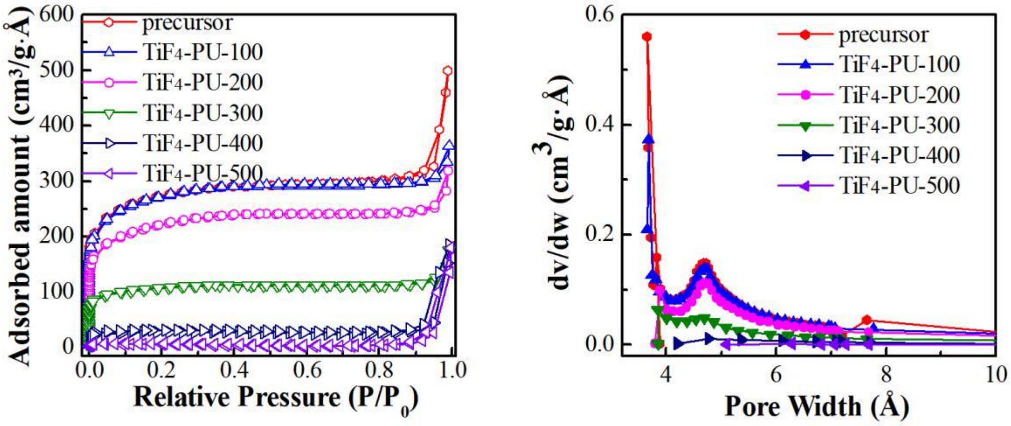

Fig. S2 BET results of TiF_4-PU composites obtained at different calcination temperatures (100, 200, 300, 400, 500°C)

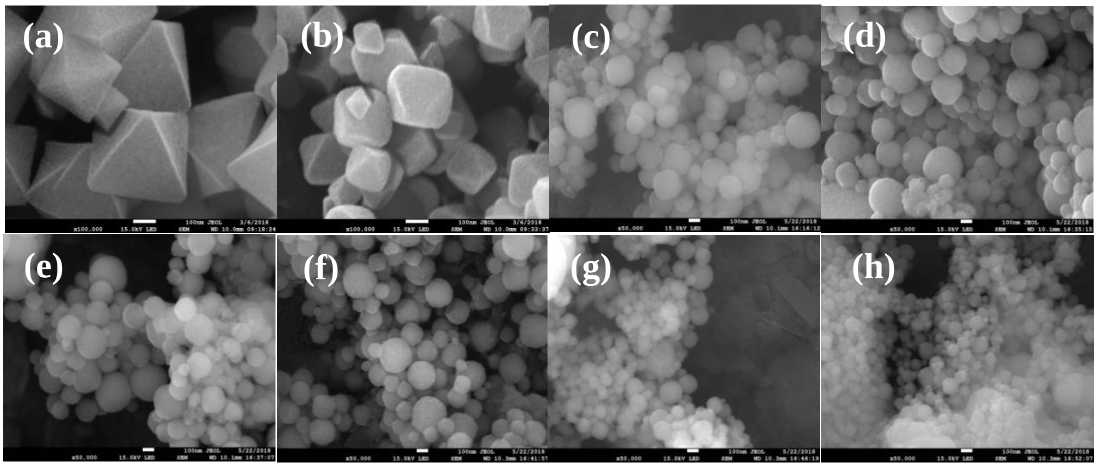

Fig. S3 SEM images: (a) UiO-66; (b) PU-200; (c)precursor;(d)TiF_4-PU-100; (e)TiF_4-PU-200;(f)TiF_4-PU-300; (g) TiF_4-PU-400; (h) TiF_4-PU -500

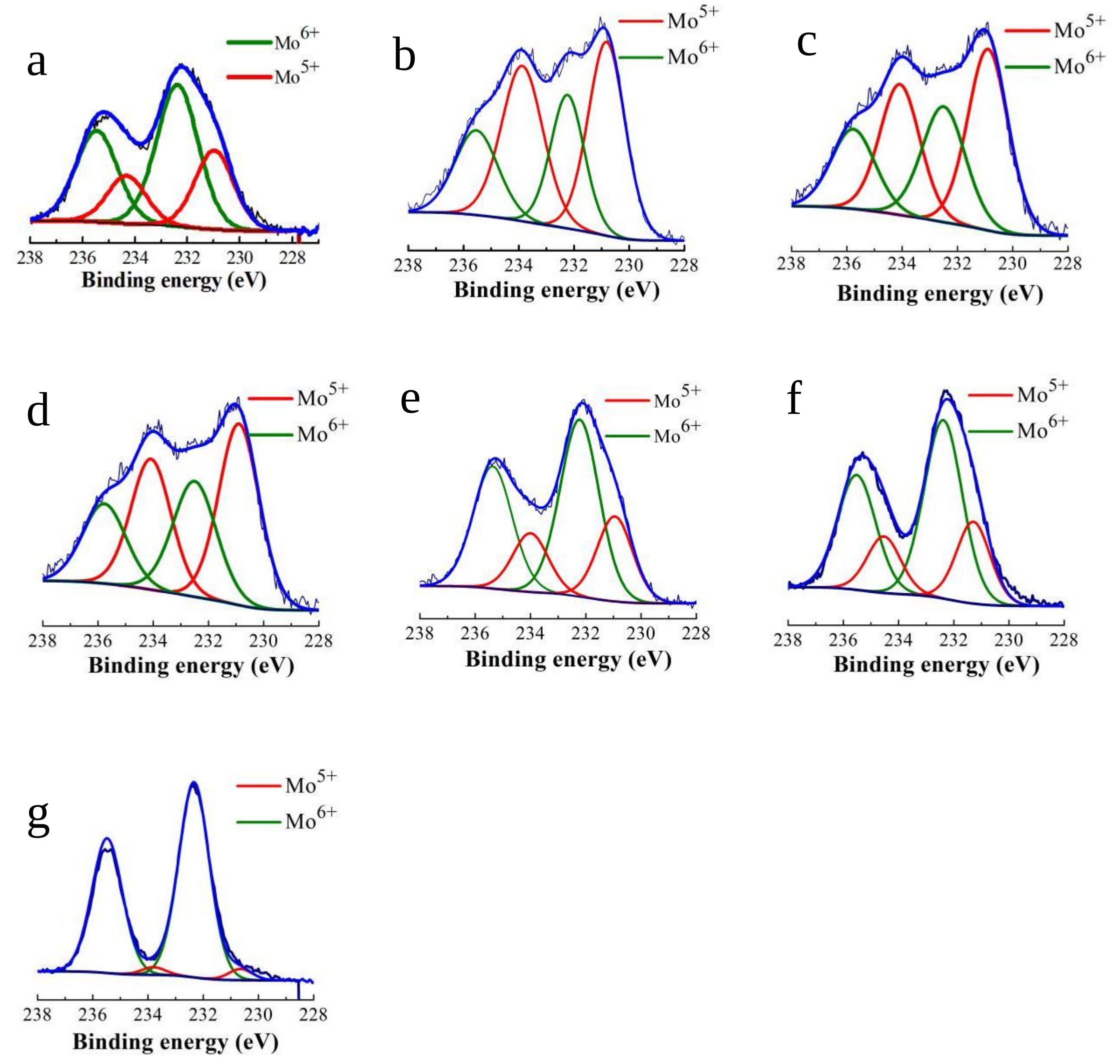

Fig. S4 XPS spectra of Mo3d: (a) PU-200; (b) precursor;(c)TiF$_4$-PU-100;(d) TiF$_4$-PU-200; (e)TiF$_4$-PU-300;　(f) Ti F$_4$-PU-400;（g）TiF$_4$-PU-500

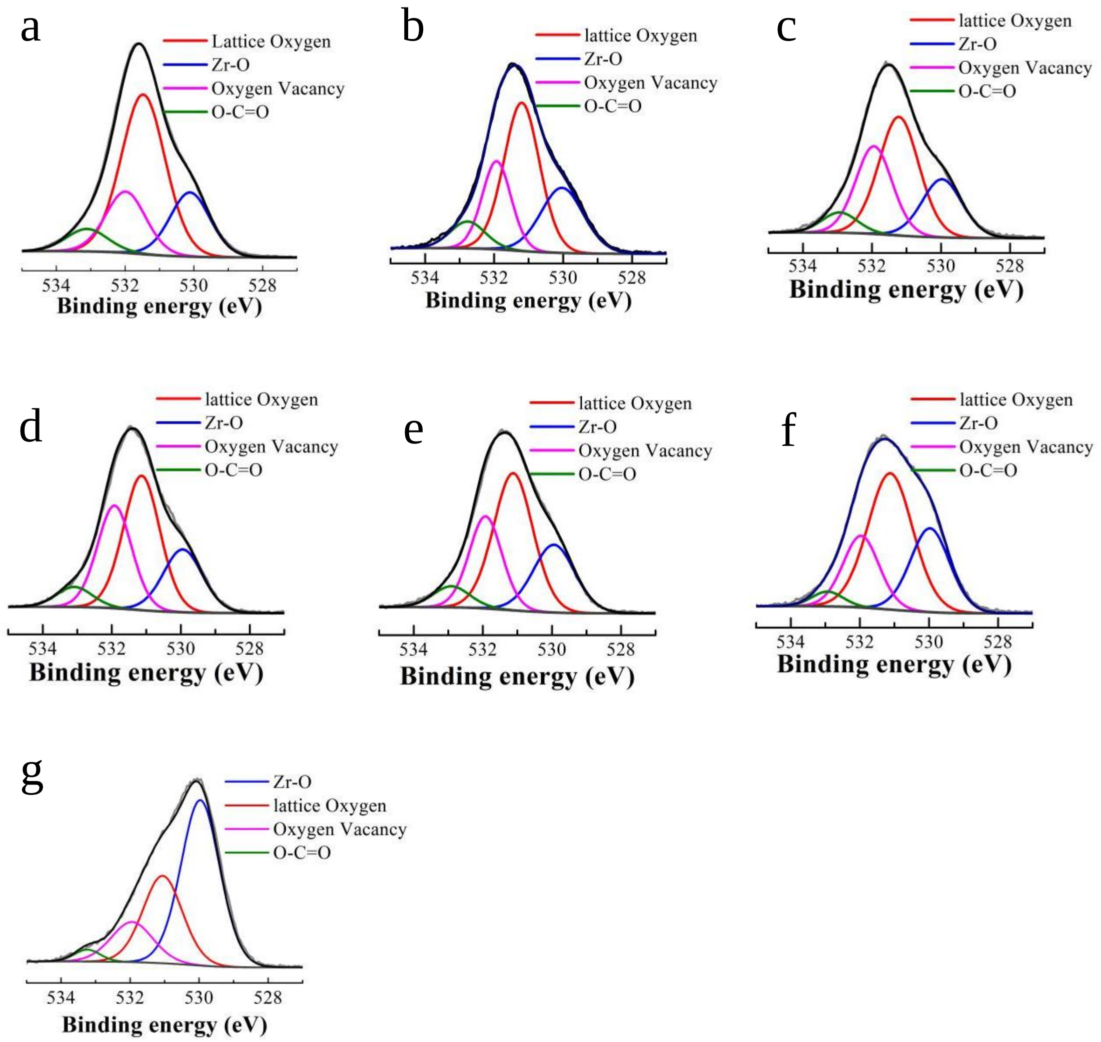

Fig. S5 XPS spectra of O_{1s}: (a) PU -200; (b) precursor;(c) TiF_4-PU-100; (d) TiF_4-PU-200; (e)TiF_4-PU-300; (f) TiF_4-PU-400; (g) TiF_4-PU-500

Table S1 ICP results of PU and TiF_4-PU catalysts.

Element	PU-200	precursor	TiF_4-PU-200
Mo	8.07	8.20	8.11
